# Incidence of asymptomatic catheter-related thrombosis in intensive care unit patients: a prospective cohort study

**DOI:** 10.1186/s13613-023-01206-w

**Published:** 2023-10-19

**Authors:** Chiara Abbruzzese, Amedeo Guzzardella, Dario Consonni, Gloria Turconi, Claudia Bonetti, Matteo Brioni, Mauro Panigada, Giacomo Grasselli

**Affiliations:** 1 Department of Anaesthesiology, Critical Care and Emergency, Intensive Care and Emergency, Fondazione IRCCS Ca’ Granda Ospedale Maggiore Policlinico, Via Francesco Sforza, 35, 20122 Milan, Italy; 2 Department of Pathophysiology and Transplantation, University of Milan, Via Francesco Sforza, 35, 20122 Milan, Italy; 3 Epidemiology Unit, Fondazione IRCCS Ca’ Granda Ospedale Maggiore Policlinico, Via Francesco Sforza, 35, 20122 Milan, Italy

**Keywords:** Asymptomatic, Catheter, Catheter-related thrombosis, Incidence rate, Risk factor

## Abstract

**Background:**

Catheter-related thrombosis (CRT) incidence, rate, and risk factors vary in literature due to differences in populations, catheters, diagnostic methods, and statistical approaches.

The aim of this single-center, prospective, observational study was to assess incidence, incidence rate (IR), cumulative incidence, and risk factors by means of IR ratio (IRR) of asymptomatic CRT in a non-oncologic Intensive Care Unit (ICU) population. CRT development was assessed daily by means of ultrasound screening. The proportions of patients and catheters developing CRT and CRT incidence rates, expressed as the number of events per catheter-days (cd), were calculated. Kalbfleisch and Prentice’s method was used to estimate the cumulative incidence of CRTs. Univariate and multivariable Poisson regression models were fitted to calculate IRR in risk factors analysis.

**Results:**

Fifty (25%, 95% CI 19–31) out of 203 included patients, and 52 (14%, 95% CI 11–18) out of 375 catheters inserted developed CRT [IR 17.7 (13.5–23.2) CRTs/1000*cd], after 5 [3–10] days from insertion. Forty-six CRTs (88%) were partial thrombosis. All CRTs remained asymptomatic. Obesity and ECMO support were patient-related protective factors [IRR 0.24 (0.10–0.60), *p* = 0.002 and 0.05 (0.01–0.50), *p* = 0.011, respectively]. The internal jugular vein had higher CRT IR than other sites [20.1 vs. 5.9 CRTs/1000*cd, IRR 4.22 (1.22–14.63), *p* = 0.023]. Pulmonary artery catheter and left-side cannulation were catheter-related risk factors [IRR 4.24 (2.00–9.00), *p* < 0.001 vs. central venous catheters; IRR 2.69 (1.45–4.98), *p* = 0.002 vs. right cannulation, respectively]. No statistically significant effect of the number of simultaneously inserted catheters [IRR 1.11 (0.64–1.94), p = 0.708] and of the catheterization length [IRR 1.09 (0.97–1.22), p = 0.155] was detected. The ICU length of stay was longer in CRT patients (20 [15–31] vs. 6 [4–14] days, *p* < 0.001), while no difference in mortality was observed.

**Conclusions:**

CRTs are frequent but rarely symptomatic. This study suggests that obesity and ECMO are protective factors, while pulmonary artery catheter, internal jugular vein and left-side positioning are risk factors for CRT.

**Supplementary Information:**

The online version contains supplementary material available at 10.1186/s13613-023-01206-w.

## Background

Insertion of a catheter in a central vein for fluid infusion and/or monitoring is required in a large proportion of patients admitted to the intensive care unit (ICU). Complications of catheter placement include catheter-related thrombosis (CRT), pulmonary embolism, catheter-related bloodstream infection, catheter malfunction, and the development of post-thrombotic syndrome [1–4].

The endothelial lesion, produced by the catheter insertion, and the consequently reduced blood flow around it lead to fibrin deposition, proliferation, and adherence of endothelial and smooth muscle cells to the catheter and vein walls, thus forming a thrombus, which can invade the lumen until the occlusion of the vein occurs [5].

Most CRTs remain subclinical [5, 6], and the literature lacks studies in which a daily screening is performed to evaluate the formation of the thrombosis, although asymptomatic. In addition, most studies focus on cancer or pediatric patients or on peripherally inserted central catheters (PICCs) [7–9]. As a result, there are still areas of uncertainty regarding the incidence, timing, and risk factors associated with CRT, which may differ from those of deep vein thrombosis. Indeed, different catheters’ positioning and characteristics could play an essential role in CRT pathogenesis [10, 11]. Therefore, we designed a prospective cohort study to determine the incidence of asymptomatic CRTs through a daily ultrasound (US) screening and to identify the possible patient and catheter-related risk factors contributing to their development.

## Methods

This is a prospective, single-center, observational cohort study on adult patients admitted to ICU from September 14th, 2020, to October 6th, 2022. Written informed consent was collected according to Italian and European regulations for clinical studies performed on critically ill patients. If patients could not provide their consent at enrollment, delayed consent was obtained. Data collected from patients who later refused consent after regaining consciousness was permanently deleted and excluded from the statistical analysis. This study was conducted following the amended Declaration of Helsinki. This study was approved by the Institutional Ethical Committee (Comitato Etico Milano Area 2 approved the study entitled “Trombosi Asintomatica Catetere Correlata: studio prospettico di coorte”, protocol n° 0010404 on March 17th, 2020) and was pre-registered at clinicaltrials.gov (NCT04503135). This study is reported according to STROBE guidelines.

Patients entered the study only when catheterized in the enrolling ICU, where the following types of catheters were used: [1] central venous catheters (CVCs, Teleflex Medical Europe, Ireland, 2–5 lumens, 20 cm, 7–8.5 Ch); [2] hemodialysis venous catheters (HDCs, Teleflex Medical Europe, Ireland, 2–3 lumens, 16–25 cm, 12–14 Ch); [3] pulmonary artery catheters (PACs, Edwards Lifesciences, California, 3 lumens, 110 cm, 7.5 Ch), positioned through an introducer (Teleflex Medical Europe, Ireland, 10 cm, 8 Ch). Patients catheterized outside the ICU environment (i.e., emergency department, general ward, operating theatre) and then admitted to ICU did not fulfill the inclusion criteria. Once a patient was enrolled in the study, any new catheter inserted was considered a new observation. The exclusion criteria for this study were: [1] age < 18 years; [2] congenital thrombophilia; [3] ongoing neoplastic disease; [4] refusal to consent. Before the insertion of any catheter, a thorough assessment of internal jugular veins (IJVs) and subclavian veins was conducted using the Rapid Central Vein Assessment (RaCeVA) protocol [12] and the compression ultrasound (CUS) of the femoral veins to evaluate all potential cannulation sites with a Philips Affiniti 70 L12-4 Transducer ultrasound (Philips, Amsterdam, Netherlands).

Every catheter was positioned by a trained second-year Intensive Care resident using real-time ultrasound guidance and under the supervision of an experienced intensivist. After the catheter placement, the correct positioning was verified via a chest X-ray, except for femoral-inserted catheters.

Ultrasound monitoring of both catheterized and not catheterized veins to check the occurrence of any thrombosis was performed daily. The study ended when one of the following conditions was met: [1] removal of the catheter (or, in case of multiple catheterizations, removal of the last remaining catheter); [2] diagnosis of catheter-related thrombosis; [3] 28 days following the last catheter insertion or patient discharge from the ICU, whichever came first; [4] patient’s death. If multiple catheterizations were performed, follow-up for all catheters ended after the first CRT was diagnosed. The follow-up of the last patient ended on October 21st, 2022.

### CRT diagnosis

Diagnosis of CRT was performed through ultrasounds, which included direct visualization of the intraluminal thrombus in combination with either one of the following criteria: [1] partial vein compressibility or partial absence of blood flow in difficult-to-compress veins (i.e., subclavian), and that was considered partial thrombosis, or [2] complete vein incompressibility or complete absence of blood flow, and that was considered complete thrombosis. Blood flow was studied using Color Doppler Flow Imaging [13–15]. Ultrasound findings were, thus, classified as: [1] no thrombosis; [2] partial thrombosis; [3] complete thrombosis.

Whenever a thrombosis was suspected during the daily US assessment performed by the intensivist, a specialistic US study was asked to a radiologist to confirm or rule out the diagnosis. Asymptomatic thrombosis was defined whenever a thrombosis was imaging-diagnosed without developing any clinical objective alteration on physical examination (i.e., tenderness, warmth, erythema or cyanosis, edema, superficial venous dilation) or without raising in the physician in charge the suspicion of pulmonary embolism.

### Catheter maintenance

The nursing staff in the ICU conducted three daily checks to ensure adherence of the medications to the vascular access site [16]. To ensure proper care of the vascular access, each lumen was kept patent by infusing maintenance fluids or using a neutral displacement clave after 10 ml of saline solution intermittent flush [17]. In addition, 4% sodium citrate was utilized as a lock solution for hemodialysis catheters when patients were not undergoing Renal Replacement Therapy [18].

### Data collection

Data were collected using REDCap electronic data capture tools hosted at Fondazione IRCCS Ca’ Granda Ospedale Maggiore Policlinico [19, 20]. During the enrollment phase, the following patient information was recorded: age, sex, Body Mass Index (BMI), admission diagnosis, Charlson Comorbidity Index, Sequential Organ Failure Assessment (SOFA) Score, diagnosis, date of ICU admission, laboratory data (hematocrit, platelets, International Normalized Ratio and activated partial thromboplastin time ratio, fibrinogen, D-dimers). Whenever, for clinical purposes, a catheter was placed, the following catheter-related data were recorded: kind of catheter inserted, catheter’s outer diameter, number of lumens, antimicrobic treatment, and catheter tip position at chest X-ray, catheterized vein and its diameter, and number of cannulation attempts. The following patient-related data were recorded daily: anticoagulant and antiplatelet therapies, need for extracorporeal membrane oxygenation (ECMO) support or surgical interventions, and results of the RaCeVa protocol and femoral veins’ CUS. Moreover, patients’ ICU outcomes and lengths of stay (LOS) were collected at the end of hospitalization.

The following cutoffs were used to differentiate prophylactic from therapeutic anticoagulation: 80 mg/day for enoxaparin, 2.5 mg/day for fondaparinux, and 200 Units/kg/day for Unfractionated Heparins. Argatroban administration was always therapeutic.

### Outcomes

The primary study outcomes were the incidence, the incidence rate, and the cumulative incidence of CRT in an ICU population. Secondary outcomes included: [1] the potential risk factors associated with CRTs; [2] the proportion of partial or complete central venous thrombosis; [3] the percentage of patients with central venous catheters who developed non-catheter-related thrombosis. We, therefore, described the clinical measures that have been taken following the diagnosis of CRT.

### Statistical analysis

Categorical variables are reported as absolute numbers and percentages. Continuous variables are presented as mean (standard deviation, SD) or median [interquartile range, IQR] for normal and non-normal distributions. Patient and catheter characteristics were described according to the CRT occurrence or not, without statistical comparisons. The proportions of patients and catheters developing CRT and 95% confidence intervals (95% CI) were calculated. Moreover, CRT incidence rates (IR) and 95% CI, expressed as events/1000 catheter-days, were calculated, considering multiple catheterizations on the same patient as different observations. Catheter cumulative incidence of CRT was estimated using the Kalbfleisch and Prentice method [21], considering death during ICU and thrombosis of another catheter as competing events.

Univariate and multivariable random-intercept Poisson regression models, with patients as random-effect, were fitted to calculate incidence rate ratios (IRR) and 95% CI of CRT. Multivariable models included admission disease and SOFA as fixed adjustment covariates; ECMO support, surgical interventions, the number of simultaneous catheters inserted on the same patient, and days of catheterization as time-dependent adjustment covariates. Analyses were performed with Stata 17 (StataCorp. 2017, Texas, USA) and JMP Pro 16 (SAS Institute Inc., Cary, NC, USA).

## Results

**Fig. 1:**
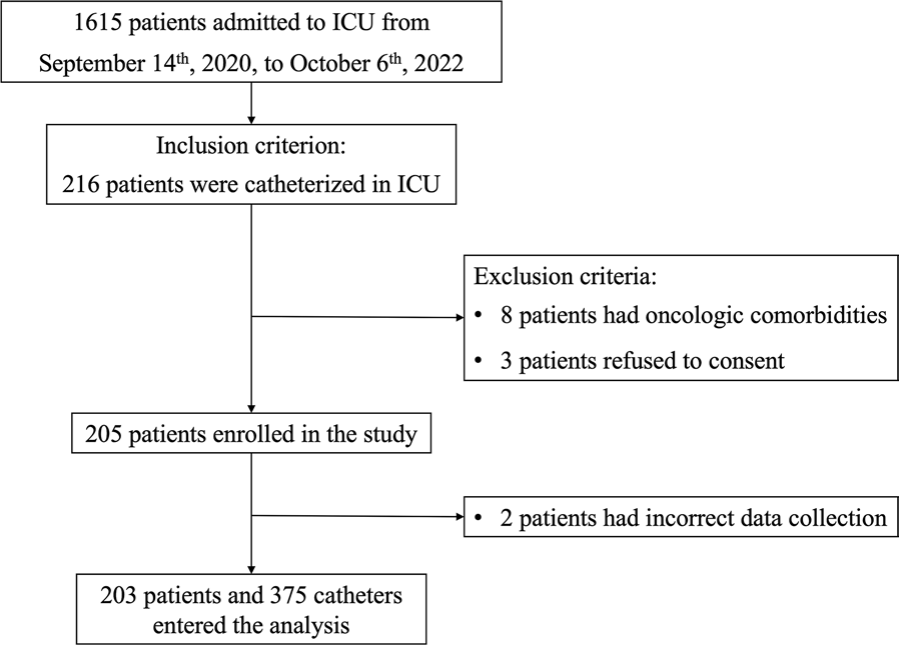
Study flowchart

During the study period, 216 out of 1615 patients needed catheterization during ICU stay and were eligible to be enrolled in the study. Three patients refused to participate, and eight patients had oncologic comorbidities. Two hundred and five patients were enrolled. Data collection was wrongly performed on two patients, and thus, they were excluded from the analysis. The remaining 203 patients entered the analysis. Enrolment occurred 0 [0–1] days after ICU admission, see Fig. 1, flowchart, for details.

One-hundred-thirty-two (65%) were males, and the median age was 60 [49–70] years. At enrollment, SOFA Score and Charlson’s Comorbidity Index were 4 [3–6] and 2 [1–4], respectively. Non-surgical diseases were the most important causes of ICU admission (178/203, 88%), and Acute Respiratory Distress Syndrome (ARDS) was the most represented diagnosis (73/203, 36%). COVID was the most represented cause of ARDS (56/73, 77%). One-hundred-five (105/203, 52%) patients were on antithrombotic prophylaxis at enrollment, whereas 80 (39%) were fully anticoagulated. One-hundred-eighteen (58%) patients needed more than one catheter during ICU stay. Twenty-one (10%) patients were treated with ECMO, and 53 (26%) underwent surgical intervention before or during ICU stay. Patients’ characteristics are presented in Table 1.

**Table 1:** Characteristics of enrolled patients

	All patients (*n* = 203)	CRT (*n* = 50)	No CRT (*n* = 153)
Age (years)	60 [49–70]	63 [52–73]	59 [49–70]
Sex (female)	71 (35%)	15 (30%)	56 (37%)
BMI (kg/m^2^)	26.1 [23.5–30.7]	25.6 [23.5–27.8]	26.1 [23.4–31.2]
SOFA score	4 [3–6]	5 [3–6]	4 [3–6]
Charlson comorbidity Index	2 [1–4]	2 [1–3]	2 [1–4]
ICU admission reason			
Medical	178 (88%)	45 (90%)	133 (87%)
ARDS COVID	56 (28%)	19 (38%)	37 (25%)
Septic shock	41 (20%)	9 (18%)	32 (21%)
ARDS	17 (8%)	6 (12%)	11 (7%)
Cardiac arrest	17 (8%)	3 (6%)	14 (9%)
Acute liver failure	7 (4%)	2 (4%)	5 (3%)
Others^a^	40 (20%)	6 (12%)	34 (22%)
Surgical	25 (12%)	5 (10%)	20 (13%)
Trauma	6 (3%)	2 (4%)	4 (3%)
Others^b^	19 (9%)	3 (6%)	16 (10%)
Laboratory data at enrollment			
Hct (%)	33 [28–38]	32 [29–38]	33 [27–38]
Platelets (10^3^/mm^3^)	202 [129–269]	199 [141–254]	207 [121–270]
INR	1.19 [1.08–1.46]	1.2 [1.07–1.48]	1.19 [1.12–1.36]
aPTT Ratio	0.95 [0.85–1.09]	0.95 [0.86–1.09]	0.94 [0.84–1.1]
Fibrinogen (mg/dL)	436 [264–643]	421 [245–642]	523 [313–657]
D-dimers (mg/dL)	3585 [1436–10476]	3585 [1357–12300]	3586 [1677–6484]
Anticoagulation during ICU stay	185 (91%)	48 (96%)	137 (90%)
Prophylaxis	105 (52%)	26 (52%)	79 (52%)
Therapy	80 (39%)	22 (44%)	58 (38%)
Antiplatelet therapy during ICU stay	43 (21%)	8 (16%)	35 (23%)
Monotherapy	32 (16%)	7 (14%)	25 (16%)
Double	11 (5%)	1 (2%)	10 (7%)
ECMO	21 (10%)	4 (8%)	17 (11%)
Surgical intervention^c^	53 (26%)	12 (24%)	41 (27%)
N. Catheters during ICU stay	2 [1, 2]	2 [1, 2]	2 [2–2]
1	85 (42%)	9 (18%)	76 (50%)
2	80 (39%)	29 (58%)	51 (33%)
3	27 (13%)	9 (18%)	18 (12%)
4	7 (3%)	2 (4%)	5 (3%)
5	3 (2%)	0 (0%)	3 (2%)
6	1 (1%)	1 (2%)	0 (0%)

*CRT* Catheter-related thrombosis, *BMI*, Body Mass Index, *SOFA*, Sequential Organ Failure Assessment; *ICU*, Intensive Care Unit; *ARDS*, Acute Respiratory Distress Syndrome; *COVID*, Coronavirus Disease; *Hct*, Hematocrit; *INR,* International Normalized Ratio; *aPTT,* activated partial thromboplastin time; *ECMO*, Extracorporeal Membrane Oxygenation

^a^Including asthma and *chronic obstructive pulmonary disease exacerbation, cardiogenic shock, diabetic ketoacidosis, hypoglycemic coma, intoxication, heat stroke, botulinum poisoning, and status epilepticus*

^b^Including lung and liver transplantation, bowel perforation and osteomyelitis

^c^Including patients admitted to ICU for surgical disease and patients who needed surgery during ICU stay for any reason

Three-hundred-seventy-five catheters were placed and followed for 2941 catheter-days (cd). Table 2 describes the catheters’ characteristics in detail.

**Table 2:** Characteristics of catheters inserted

		All catheters (*n* = 375)	CRT (*n* = 52)	No CRT (*n* = 323)
Type	*n* = 375			
Central venous catheter		266 (71%)	32 (62%)	234 (72%)
Pulmonary artery catheter		63 (17%)	18 (35%)	45 (14%)
Hemodialysis catheter		46 (12%)	2 (3%)	44 (14%)
Side	*n* = 375			
Right		255 (68%)	25 (48%)	230 (71%)
Left		120 (32%)	27 (52%)	93 (29%)
Site				
Internal jugular vein		302 (81%)	49 (94%)	253 (78%)
Femoral vein		60 (16%)	2 (4%)	58 (18%)
Subclavian and axillary veins		13 (3%)	1 (2%)	12 (4%)
N. of lumen	*n* = 351			
2		15 (4%)	2 (4%)	13 (4%)
3		206 (59%)	29 (59%)	177 (59%)
4		80 (23%)	11 (22%)	69 (23%)
5		50 (14%)	7 (15%)	43 (14%)
Antimicrobial coated catheter	*n* = 337	86 (26%)	12 (25%)	74 (26%)
Catheter’s tip X-ray position^a^	*n* = 278/315 §			
CVC and hemodialysis catheter	*n* = 224/252			
RA		19 (8%)	4 (15%)	15 (8%)
SVC–RA junction		44 (20%)	5 (19%)	39 (20%)
SVC		161 (72%)	18 (66%)	143 (72%)
Pulmonary artery catheter	*n *= 54/63			
Right PA		30 (56%)	7 (43%)	23 (60%)
Left PA		9 (17%)	3 (19%)	6 (16%)
Pulmonary trunk		15 (27%)	6 (38%)	9 (24%)
N. cannulation attempts	*n* = 322			
1		297 (92%)	41 (91%)	256 (93%)
> 1		25 (8%)	4 (9%)	21 (7%)
Catheter/vein diameter ratio	*n* = 270	0.21 [0.16–0.25]	0.22 [0.16–0.25]	0.21 [0.16–0.26]
< 0.3		237 (88%)	38 (90%)	200 (88%)
≥ 0.3		32 (12%)	4 (10%)	28 (12%)

*CVC* central venous catheter, *RA* right atrium, *SVC *superior vena cava, *PA *pulmonary artery

^a^315 catheters included [Excluding catheters inserted in the femoral vein (*n* = 60)]

The median duration of catheter follow-up was 5 [3–10] days. CVCs (266, 71%) were the most frequently positioned, followed by PACs (63, 17%) and HDCs (46, 12%). The most common site of cannulation was the IJV (302, 81%). In total, 255 (68%) catheters were positioned on the right side of the body, of which 206 (81%) were in the right IJV. Additional file 1: Table S3 reports details of the type, site, and side of cannulation divided by catheters with and without CRT. More than one cannulation attempt was performed 25 times (7%). Catheter–vein diameter ratio was 0.21 [0.16–0.25].

**Fig. 2:**
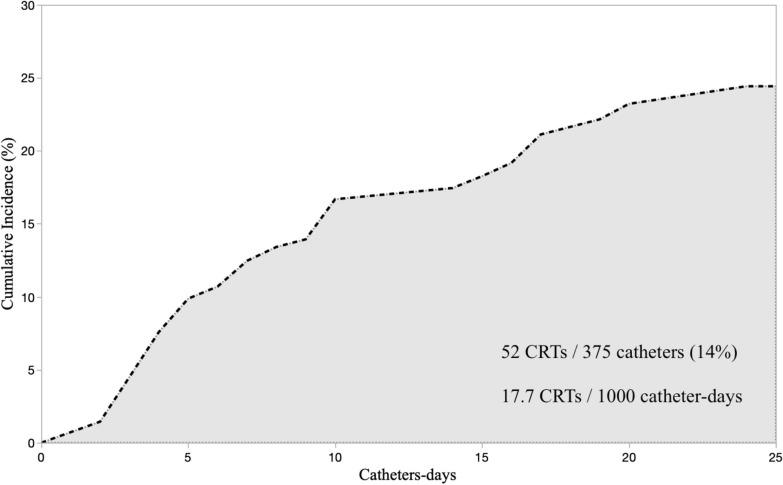
Cumulative incidence of catheter-related thrombosis

Fifty out of 203 (25%, 95% CI 19–31) patients developed a CRT. Two patients had CRTs on two distinct catheters on the same day. The proportion of CRTs was 52 out of 375 (14%, 95% CI 11–18), and the incidence rate (95% CI) was 17.7 (13.5–23.2) CRTs/1000*cd. Figure 2 shows the catheters' cumulative incidence of CRT. The median time interval between catheter placement and CRT was 5 [3–10] days. Forty-six CRTs (88%) were partial thrombosis, while only 6/52 (12%) were complete thrombosis. Notably, no CRT became symptomatic.

Patients with BMI ≥ 30 and patients who needed ECMO support had lower IR of CRT [6.3 vs. 23.1 CRTs/1000*cd (adjusted IRR 0.24 (0.10–0.60), *p* = 0.002) and 1.9 vs. 21.2 CRTs/1000*cd (adjusted IRR 0.05 (0.01–0.50), *p* = 0.011), respectively]. Neither the number of simultaneously inserted catheters nor the duration of catheterization itself statistically increased the risk of developing CRTs [adjusted IRR for one unit increment: 1.11 (0.64–1.94), p = 0.708; and 1.09 (0.97–1.22), p = 0.155, respectively]. No other patient characteristic impacted CRT IR. Additional file 2: Table S4 shows patient-related risk factors analysis.

Catheter characteristics associated with a higher incidence of CRT were the type of catheter [PACs had adjusted IRR of 4.24 (2.00–9.00), *p* < 0.001 vs. CVC] and left side cannulation [adjusted IRR 2.69 (1.45–4.98), *p* = 0.002 vs. right cannulation]. Internal jugular vein cannulation had higher CRT IR than other sites [20.1 vs. 5.9 CRTs/1000*cd, adjusted IRR 4.22 (1.22–14.63), *p* = 0.023]. No other catheter characteristic had a statistically significant effect on CRT development. Additional file 3: Table S5 shows catheter-related risk factors analysis.

Five adverse events were reported during catheter positioning: four hematomas and one accidental carotid artery puncture. One catheter needed immediate replacement after X-rays showing contralateral anonymous vein tip placement. No iatrogenic pneumothorax was registered.

Eighteen (36%) patients were on therapeutic anticoagulation at CRT diagnosis. Seventeen (34%) patients started anticoagulation after CRT diagnosis, whereas in 15 patients (30%), no clinical changes in therapy were decided by the ICU doctor in charge. Where in doubt, the decision to anticoagulate the patient was taken jointly by the intensivist and the hematologist. In Additional file 4: Table S6, the characteristics of these two sub-cohorts of patients are described. No statistical differences between other variables were found except for non-COVID ARDS as the admission reason. None of the catheters malfunctioned due to CRT; thus, none was immediately removed. Neither bleeding related to the start of anticoagulation therapy nor thrombotic complications in not treated patients were observed.

During the study period, nine patients (4%) developed a deep venous thrombosis in a non-catheterized vein.

Mortality in ICU did not differ between patients who suffered vs. those who did not suffer from CRT (23% vs. 30%, *p* = 0.323), while ICU LOS was longer in CRT patients (20 [15–31] vs. 6 [4–14], p < 0.001). The ICU LOS in CRT patients is primarily due to time spent in the ICU after CRT development rather than before (days between ICU admission and CRT 6 [3–11] vs. days between CRT and ICU outcome 12 [4–21]).

## Discussion

The main finding of the study was that a daily ultrasound screening revealed a high proportion of asymptomatic CRTs: 25% of enrolled patients and 17% of the catheters developed CRT, corresponding to an incidence rate of 17.7 CRTs/1000*cd. Of note, none of the 52 CRTs detected when asymptomatic became later symptomatic. We also identified some catheter-related characteristics as risk factors for CRT (catheter type and catheter site and side) and some patient-related ones as protective factors (BMI and ECMO treatment). Furthermore, we highlighted that neither the number of simultaneous inserted catheters nor the duration of catheterization itself are risk factors for CRT. Finally, we described the anticoagulation management post-CRT diagnosis decided by the physicians in charge.

Previous literature describing CRT incidence is available, but results vary significantly due to different study designs (retrospective vs. prospective), catheter selection (PICCs [22] vs. CVCs [23]), population selection (pediatric [24] vs. adult patients or only-cancer [25] vs. non-cancer patients), and CRT diagnostic method (venography [26] vs. US.). Timsit et al. [4], in a prospective multicentric study conducted in three medical–surgical ICUs, observed a higher proportion of CRT (33% of 208 patients) than what we found. Some important differences in the study design can explain this discrepancy. First, their population included cancer patients. Second, improvement in catheter materials technology (i.e., less thrombogenic) could have lowered the risk of CRTs in our population [9]. Third, we performed US-guided catheterization, and this could have allowed us to reduce the number of catheter positioning attempts.

Gunther et al. [23] reported a higher incidence rate of catheter thrombosis (33 CRTs/1000*cd). Still, again, they included cancer patients and studied both arterial and central venous catheters without reporting disaggregated data from the various catheter types.

Recently, Wu et al. [27], in a prospective, multicenter study, including 1262 patients, reported a CRT in 16.9% of patients. Similar to us, they performed a daily US assessment to detect CRT formation before any symptoms developed. Some relevant differences between the studies should be noted. In particular, they did not include HDCs and PACs, and the sites of catheterization were different, with a lower proportion of catheters placed in the IJV (54% vs. 81%) and a higher rate of subclavian catheterization (30% vs. 2%). Interestingly, none of their CRT was symptomatic.

Female sex, older age, and higher BMI are commonly considered risk factors for venous thrombosis [28, 29]. However, all previous studies evaluated risk factors using odds (and odds ratios) of developing CRTs, thus neglecting time at risk. We did consider time by calculating incidence rates and found no association between CRT occurrence and sex; age ≥ 65 years had a 30% higher CRT rate (although with wide CIs), and, surprisingly, obesity was associated with markedly lower CRT incidence rate. All these patient’s characteristics could affect the duration of ICU length-of-stay [30, 31]. Therefore, we think that IRR rather than the odds ratio may be more correct in describing CRT’s risk and protective factors. This could be why our risk factors analysis results are slightly different from previous literature. Indeed, despite the median BMI of the two cohorts of patients being similar (25.6 [23.5–27.8] kg/m^2^ in CRT patients vs. 26.1 [23.4–31.2] kg/m^2^ in no CRT patients), our signal in the analysis comparing IR is very strong: the higher the BMI, the slower the thrombus formation. However, the novelty of this result surely deserves further studies to be confirmed.

In our study, the most frequent diagnosis of admission was COVID-19 ARDS. Although the demonstrated hypercoagulability of COVID patients [32], the disease was not associated with a higher risk of CRT in our patient. This could be due to the enhanced anticoagulation regimen adopted in our institution for this disease. Anticoagulation was not highlighted as a protective factor. Nevertheless, we think the lower incidence rate of CRT in ECMO patients could be traced back to the strict anticoagulation monitoring of these patients, which, in our center, is performed at least three times a day.

PACs had a higher rate of CRTs rather than CVCs and HDCs. We did not find any study evaluating this kind of catheter specifically. Still, we can hypothesize that the need for a large introducer [33], our preference for IJV positioning, and the higher length of the catheter are possible explanations for this result. Moreover, we observed lower CRTs in the femoral rather than the IJV approach and left-side positioning as a risk factor for CRT. These results are consistent with previously published studies [34–36].

The employment of US guidance during each catheter positioning maneuver may have contributed to the observed low incidence of adverse events and their relatively mild severity [37].

We described heterogeneous therapeutic management of patients after CRT diagnosis, and we failed to identify any patient characteristic that could have guided the physician's decision to start full anticoagulation, except for ARDS as the admission diagnosis. Deciding to start anticoagulation, for how long, and with which drug are still topics of research and discussion in symptomatic CRT, especially those of upper extremities [38]. CHEST guidelines [39] suggested not removing the catheter if functional, starting anticoagulation, and continuing it at least 3 months after its removal. The grading of this recommendation is a 2C. Of note, we did not observe any adverse events due to the choice of not starting anticoagulation, and, according to Wu et al. [27], most of their CRTs regress spontaneously without it. Our study and Wu’s [27], taken together, account for a total of 263 patients who developed asymptomatic CRTs. Even without a specific daily screening, in routine clinical settings, some of them could have been diagnosed with CRT incidentally and thus, according to guidelines, anticoagulated for months. Future research should investigate if, in these cases, applying the same guidelines of symptomatic thrombosis is suitable.

Similar to the results of Wu [27], our patients who suffered from CRT had longer ICU stays but no mortality difference. At first glance, explaining this with a higher risk of CRT in long-lasting hospitalized patients may be tempting. Actually, CRT patients spent much time hospitalized after CRT diagnosis, not before. Since asymptomatic CRTs are unlikely to cause longer hospitalization, we can speculate that they are, at least, an early sign of a possible long ICU stay.

Our study has several strengths. The most important is the daily assessment of CRT, which allowed us to promptly detect thrombosis, even if asymptomatic. We were able to report the estimates of a time-dependent event as incidence rate rather than proportion. Second, we considered a heterogeneous population with different kinds of catheters inserted, which may provide us with the information currently lacking in the scientific literature. Third, we asked a radiology physician to confirm or rule out every doubt or suspected CRT identified during the ICU physician's daily assessment, to increase our accuracy in CRT diagnosis [40].

Our study has several limitations, too. First, we censored follow-up at patient ICU discharge. Thus, we cannot account for CRTs that could have developed later. Second, contrary to other studies [27, 34], in which US screening was continued for 2 days after catheter removal or after CRT, we did not plan to continue with a US follow-up, and we were not able to describe the evolution of CRTs in anticoagulated vs. not anticoagulated patients. Third, we evaluated asymptomatic thrombosis on behalf of clinical objective alteration at physical examination. Thus, we cannot exclude that some unconscious patients were subjectively symptomatic. Fourth, the habits of our ICU physicians (i.e., use of PAC as gold-standard hemodynamic monitoring, low rate of subclavian CVCs positioning), the choice to include only patients catheterized in the ICU and not in other hospital settings, and the monocentric nature of our study may limit the external validity of our results. Finally, we still have many unanswered questions regarding other possible risk factors for CRT, such as whether different US puncture techniques (i.e., long axis vs. short axis or in-plane vs. out-of-plane approach), catheter-related infections, or even catheter’s depth of insertion could affect the development of CRTs [41].

## Conclusions

In our patient population, CRTs were frequent, rarely symptomatic, and most were partial thrombosis. The kind and the site of the catheter could play a role in favoring or protecting from CRT. Obesity and ECMO treatment were protective factors. Further studies are required to define the clinical impact of CRTs and should investigate the best coagulation management after CRT diagnosis.

### Supplementary Information

**Additional file 1: Table S3.** Site, type and side of catheter studied, divided by CRT and no-CRT catheters.

**Additional file 2: Table S4.** Patient-related risk factors for CRT analysis

**Additional file 3: Table S5.** Catheter-related risk factors for CRT analysis.

**Additional file 4: Table S6.** Characteristics of CRT patients divided by patients for whom the physician decided to start anticoagulation vs. patients not anticoagulated.

## Data Availability

The data sets used and/or analysed during the current study are available from the corresponding author upon reasonable request.

## Abbreviations

ARDS: Acute respiratory distress syndrome
BMI: Body mass index
cd: Catheter-days
CI: Confidence interval
CRT: Catheter-related thrombosis
CUS: Compression ultrasound
CVC: Central venous catheter
ECMO: Extracorporeal membrane oxygenation
HDC: Hemodialysis venous catheter
ICU: Intensive care unit
IJV: Internal jugular vein
IQR: Interquartile range
IR: Incidence rate
IRR: Incidence rate ratio
LOS: Length of stay
PAC: Pulmonary artery catheter
pd: Patient-day
PICC: Peripherally inserted central catheter
RA: Right atrium
RaCeVA: Rapid central vein assessment
SD: Standard deviation
SOFA: Sequential organ failure assessment
US: Ultrasound
