# Supplementary material for: Incidence of asymptomatic catheter-related thrombosis in intensive care unit patients: a prospective cohort study
Source: Ann Intensive Care. 2023 Oct 19;13:106. doi: 10.1186/s13613-023-01206-w (PMC10587047; doi:10.1186/s13613-023-01206-w)
Supplement: Supplementary file 1 — Additional file 1: Table S3. Site, type and side of catheter studied, divided by CRT and no-CRT catheters. [file 13613_2023_1206_MOESM1_ESM.docx]

|  | Overall  (n=375) | | | CRT catheters  (n=52, 14%) | | | NO CRT catheters  (n=323, 86%) | | |
| --- | --- | --- | --- | --- | --- | --- | --- | --- | --- |
| **Catheter Site, Type** | **Overall (n=375)** | **Right side (n=255)** | **Left side (n=120)** | **Overall (n=52)** | **Right side (n=25)** | **Left side (n=27)** | **Overall (n=323)** | **Right side (n=230)** | **Left side (n=93)** |
| Jugular Internal vein, n. (%) | 302 (81%) | 206 (81%) | 96 (80%) | 49 (94%) | 23 (92%) | 26 (96%) | 253 (78%) | 183 (80%) | 70 (75%) |
| Central Venous Catheter, n. (%) | 224 (60%) | 168 (66%) | 56 (47%) | 29 (56%) | 20 (80%) | 9 (33%) | 195 (60%) | 148 (64%) | 47 (51%) |
| Hemodialysis Catheter, n. (%) | 15 (4%) | 6 (2%) | 9 (8%) | 2 (4%) | 0 (0%) | 2 (7%) | 13 (4%) | 6 (3%) | 7 (8%) |
| Pulmonary Artery Catheter, n. (%) | 63 (17%) | 32 (13%) | 31 (26%) | 18 (35%) | 3 (12%) | 15 (56%) | 45 (14%) | 29 (13%) | 16 (17%) |
| Femoral vein, n. (%) | 60 (16%) | 39 (15%) | 21 (18%) | 2 (4%) | 1 (4%) | 1 (4%) | 58 (18%) | 38 (17%) | 20 (22%) |
| Central Venous Catheter, n. (%) | 29 (8%) | 20 (8%) | 9 (8%) | 2 (4%) | 1 (4%) | 1 (4%) | 27 (8%) | 19 (8%) | 8 (9%) |
| Hemodialysis Catheter, n. (%) | 31 (8%) | 19 (7%) | 12 (10%) | 0 (0%) | 0 (0%) | 0 (0%) | 31 (10%) | 19 (8%) | 12 (13%) |
| Subclavian and Axillary veins, n. (%) | 13 (3%) | 10 (4%) | 3 (3%) | 1 (2%) | 1 (4%) | 0 (0%) | 12 (4%) | 9 (4%) | 3 (3%) |
| Central Venous Catheter, n. (%) | 13 (3%) | 10 (4%) | 3 (3%) | 1 (2%) | 1 (4%) | 0 (0%) | 12 (4%) | 9 (4%) | 3 (3%) |

**Table 3.**

Site, type and side of catheter studied, divided by CRT and NO-CRT catheters.

*ECMO*, Extracorporeal Membrane Oxygenation
