# Supplementary material for: Incidence of asymptomatic catheter-related thrombosis in intensive care unit patients: a prospective cohort study
Source: Ann Intensive Care. 2023 Oct 19;13:106. doi: 10.1186/s13613-023-01206-w (PMC10587047; doi:10.1186/s13613-023-01206-w)
Supplement: Supplementary file 2 — Additional file 2: Table S4. Patient-related risk factors for CRT analysis [file 13613_2023_1206_MOESM2_ESM.docx]

**Table 4**

Patient-related risk factors for CRT analysis.

|  |  |  |  | Unadjusted | |  | Adjusted ^a^ | |
| --- | --- | --- | --- | --- | --- | --- | --- | --- |
|  | CRT  (n. events) | Days at risk  (cd) | IR  (events/1000*cd) | IRR (95% CI) | *P-value* |  | IRR (95% CI) | *P-value* |
| Age (years) | - | - | - | 1.01 (0.98 – 1.03) ^b^ | 0.678 |  | 1.01 (0.98 – 1.03) ^b^ | 0.483 |
| < 65 years | 28 | 1771 | 15.8 | 1.00 (reference) |  |  | 1.00 (reference) |  |
| ≥ 65 years | 24 | 1170 | 20.5 | 1.28 (0.68 – 2.44) | 0.447 |  | 1.39 (0.67 – 2.92) | 0.379 |
| Sex |  |  |  |  |  |  |  |  |
| Male | 35 | 2007 | 17.4 | 1.00 (reference) |  |  | 1.00 (reference) |  |
| Female | 17 | 934 | 18.2 | 1.00 (0.51 – 1.97) | 0.993 |  | 0.92 (0.44 – 1.95) | 0.838 |
| BMI (kg/m^2^) | - | - | - | 0.93 (0.87 – 0.98) ^b^ | 0.014 |  | 0.91 (0.86 – 0.97) ^b^ | 0.005 |
| < 30 | 46 | 1988 | 23.1 | 1.00 (reference) |  |  | 1.00 (reference) |  |
| ≥ 30 | 6 | 953 | 6.3 | 0.26 (0.11 – 0.64) | 0.003 |  | 0.24 (0.10 – 0.60) | 0.002 |
| SOFA Score | - | - | - | 0.90 (0.80 – 1.02) ^b^ | 0.102 |  | 0.90 (0.78 – 1.05) ^b^ | 0.177 |
| < 7 | 43 | 2199 | 19.5 | 1.00 (reference) |  |  | 1.00 (reference) |  |
| ≥ 7 | 9 | 742 | 12.1 | 0.63 (0.29 – 1.34) | 0.228 |  | 0.67 (0.27 – 1.68) | 0.398 |
| ICU Admission Reason |  |  |  |  |  |  |  |  |
| Medical | 46 | 2742 | 16.7 | 1.00 (reference) |  |  | 1.00 (reference) |  |
| Surgical | 6 | 199 | 30.2 | 1.47 (0.55 – 3.89) | 0.439 |  | 0.76 (0.11- 5.08) | 0.774 |
| Disease at admission |  |  |  |  |  |  |  |  |
| Others ^c^ | 10 | 487 | 20.5 | 1.00 (reference) |  |  | 1.00 (reference) |  |
| ARDS COVID | 19 | 1266 | 15.0 | 0.84 (0.34 - 2.06) | 0.704 |  | 1.13 (0.35 - 3.59) | 0.842 |
| Septic Shock | 9 | 679 | 13.3 | 0.68 (0.25 - 1.81) | 0.439 |  | 0.66 (0.21 - 2.11) | 0.487 |
| ARDS | 6 | 186 | 32.2 | 1.61 (0.53 - 4.89) | 0.401 |  | 2.20 (0.57 - 8.48) | 0.251 |
| Cardiac Arrest | 3 | 207 | 14.5 | 0.73 (0.19 - 2.84) | 0.644 |  | 0.94 (0.20 - 4.44) | 0.940 |
| Acute Liver Failure | 3 | 65 | 46.2 | 2.09 (0.49 - 8.85) | 0.317 |  | 2.32 (0.40 - 13.29) | 0.345 |
| Trauma | 2 | 51 | 39.2 | 1.96 (0.38 - 10.14) | 0.422 |  | 1.73 (0.25 - 12.10) | 0.580 |
| Anticoagulation |  |  |  |  |  |  |  |  |
| Prophylaxis | 31 | 1382 | 22.4 | 1.00 (reference) |  |  | 1.00 (reference) |  |
| Therapy | 17 | 1228 | 13.8 | 0.67 (0.34 - 1.31) | 0.241 |  | 1.22 (0.56 - 2.63) | 0.619 |
| None | 4 | 331 | 12.1 | 0.48 (0.16 - 1.41) | 0.184 |  | 0.50 (0.16 - 1.59) | 0.241 |
| Antiplatelet therapy |  |  |  |  |  |  |  |  |
| None | 45 | 2543 | 17.7 | 1.00 (reference) |  |  | 1.00 (reference) |  |
| Mono | 6 | 295 | 20.3 | 1.07 (0.42 - 2.73) | 0.893 |  | 0.77 (0.26 - 2.33) | 0.647 |
| Dual | 1 | 103 | 9.7 | 0.55 (0.07 - 4.47) | 0.573 |  | 0.62 (0.06 - 6.65) | 0.694 |
| ECMO |  |  |  |  |  |  |  |  |
| No | 51 | 2407 | 21.2 | 1.00 (reference) |  |  | 1.00 (reference) |  |
| Yes | 1 | 534 | 1.9 | 0.08 (0.01 - 0.59) | 0.013 |  | 0.05 (0.01 - 0.50) | 0.011 |
| Surgery ^d^ |  |  |  |  |  |  |  |  |
| No | 38 | 2368 | 16 | 1.00 (reference) |  |  | 1.00 (reference) |  |
| Yes | 14 | 573 | 24.4 | 1.47 (0.73 – 2.98) | 0.280 |  | 1.37 (0.51 – 3.66) | 0.536 |
| N. Catheters inserted at the same time |  |  |  | 0.89 (0.57 – 1.40) ^b^ | 0.623 |  | 1.11 (0.64 – 1.94) ^b^ | 0.708 |
| 1 | 18 | 943 | 19.1 | 1.00 (reference) |  |  | 1.00 (reference) |  |
| 2 | 28 | 1530 | 18.3 | 1.10 (0.57 – 2.11) | 0.785 |  | 1.73 (0.83 – 3.62) | 0.143 |
| 3 | 6 | 452 | 13.3 | 0.72 (0.26 – 2.03) | 0.536 |  | 1.23 (0.35 – 4.41) | 0.746 |
| 4 | 0 | 16 | 0.0 | - | - |  | - | - |
| Days of catheterization |  |  |  | 1.07 (0.94 – 1.22) ^b^ | 0.315 |  | 1.09 (0.97 – 1.22) ^b^ | 0.155 |

^a^ Adjusted for: admission disease, SOFA Score at enrollment, as fixed coviariates; ECMO, Surgery, number of catheters inserted on the same patient and days of catheterization as time-varying covariate.

^b^ For 1 unit increment.

^c^ Including asthma and chronic obstructive pulmonary disease exacerbation, cardiogenic shock, diabetic ketoacidosis, hypoglycemic coma, intoxication, heat stroke, botulinum poisoning and status epilepticus, lung and liver transplantation, bowel perforation and osteomyelitis.

^d^ Including patients admitted to ICU for surgical disease and patients who needed surgery during ICU stay for any reason.

*CRT*, Catheter-Related Thrombosis, *IR,* Incidence Rate; *IRR,* Incidence Rate Ratio; *CI*, Confidence Interval; *pd*, patient-days; *BMI*, Body Mass Index, *SOFA*, Sequential Organ Failure Assessment; *ICU*, Intensive Care Unit; *ARDS*, Acute Respiratory Distress Syndrome; *COVID*, Coronavirus Disease; *ECMO*, Extracorporeal Membrane Oxygenation.
