# Supplementary material for: Incidence of asymptomatic catheter-related thrombosis in intensive care unit patients: a prospective cohort study
Source: Ann Intensive Care. 2023 Oct 19;13:106. doi: 10.1186/s13613-023-01206-w (PMC10587047; doi:10.1186/s13613-023-01206-w)
Supplement: Supplementary file 3 — Additional file 3: Table S5. Catheter-related risk factors for CRT analysis. [file 13613_2023_1206_MOESM3_ESM.docx]

**Table 5**

Catheter-related risk factors for CRT analysis.

|  |  |  |  | Unadjusted | |  | Adjusted^a^ | |
| --- | --- | --- | --- | --- | --- | --- | --- | --- |
|  | CRT  (n. events) | Days at risk  (catheter-days) | IR  (events/1000*cd) | IRR | *P-value* |  | IRR | *P-value* |
| Catheter Type |  |  |  |  |  |  |  |  |
| Central Venous Catheter | 32 | 2007 | 15.9 | 1.00 (reference) |  |  | 1.00 (reference) |  |
| Pulmonary Artery Catheter | 18 | 553 | 32.5 | 2.30 (1.23 - 4.27) | 0.009 |  | 4.24 (2.00 – 9.00) | < 0.001 |
| Hemodialysis Catheter | 2 | 381 | 5.2 | 0.33 (0.08 - 1.43) | 0.141 |  | 0.36 (0.08 - 1.56) | 0.172 |
| Catheter Side |  |  |  |  |  |  |  |  |
| Right | 25 | 1870 | 13.4 | 1.00 (reference) |  |  | 1.00 (reference) |  |
| Left | 27 | 1071 | 25.2 | 2.24 (1.24 – 4.07) | 0.008 |  | 2.69 (1.45 – 4.98) | 0.002 |
| Catheter Site |  |  |  |  |  |  |  |  |
| Jugular Internal vein | 49 | 2434 | 20.1 | 1.00 (reference) |  |  | 1.00 (reference) |  |
| Femoral vein | 2 | 409 | 4.9 | 0.23 (0.05 – 0.97) | 0.045 |  | 0.21 (0.05 – 0.89) | 0.035 |
| Subclavian and Axillary veins | 1 | 98 | 10.2 | 0.44 (0.05 - 3.51) | 0.436 |  | 0.35 (0.04 – 2.93) | 0.331 |
| Catheter’s N. of Lumen |  |  |  | 0.83 (0.57 – 1.21)^b^ | 0.329 |  | 0.78 (0.50 – 1.20) ^b^ | 0.255 |
| 2 | 2 | 92 | 21.7 | 1.00 (reference) |  |  | 1.00 (reference) |  |
| 3 | 29 | 1474 | 19.7 | 1.00 (0.22 - 4.53) | 0.998 |  | 0.98 (0.2 - 4.74) | 0.980 |
| 4 | 11 | 642 | 17.1 | 0.79 (0.16 - 3.89) | 0.771 |  | 0.65 (0.11 - 3.76) | 0.630 |
| 5 | 7 | 575 | 12.2 | 0.67 (0.13 - 3.53) | 0.637 |  | 0.59 (0.10 - 3.56) | 0.567 |
| Antimicrobial Coated Catheter |  |  |  |  |  |  |  |  |
| No | 36 | 2136 | 16.9 | 1.00 (reference) |  |  | 1.00 (reference) |  |
| Yes | 12 | 597 | 20.1 | 1.08 (0.53 – 2.21) | 0.825 |  | 1.14 (0.53 – 2.44) | 0.737 |
| Catheter’s tip RX position§ |  |  |  |  |  |  |  |  |
| CVC and Hemodialysis catheter |  |  |  |  |  |  |  |  |
| SVC – RA Junction | 5 | 379 | 13.2 | 1.00 (reference) |  |  | 1.00 (reference) |  |
| SVC | 18 | 1319 | 13.6 | 1.07 (0.38 – 3.00) | 0.900 |  | 1.38 (0.49 – 3.85) | 0.541 |
| RA | 4 | 119 | 33.6 | 2.78 (0.67 – 11.48) | 0.157 |  | 2.93 (0.76 – 11.27) | 0.119 |
| Pulmonary Artery Catheter |  |  |  |  |  |  |  |  |
| Right PA | 7 | 306 | 22.9 | 1.00 (reference) |  |  | 1.00 (reference) |  |
| Left PA | 3 | 79 | 38 | 1.60 (0.36 – 7.20) | 0.540 |  | 0.61 (0.12 – 3.16) | 0.556 |
| Pulmonary Trunk | 6 | 87 | 69 | 2.97 (0.89 – 9.85) | 0.076 |  | 1.03 (0.29 – 3.69) | 0.960 |
| Catheterization n. attempts |  |  |  | 0.82 (0.32 – 2.16) ^b^ | 0.707 |  | 0.80 (0.27 – 2.42) ^b^ | 0.697 |
| 1 | 41 | 2431 | 16.9 | 1.00 (reference) |  |  | 1.00 (reference) |  |
| > 1 | 4 | 213 | 18.8 | 0.98 (0.32 – 3.03) | 0.973 |  | 0.92 (0.25 – 3.36) | 0.902 |
| Catheter/Vein Diameter Ratio (n=270) |  |  |  | 3.64 (0.13 – 102.29) ^b^ | 0.447 |  | 1.18 (0.04 – 36.66) ^b^ | 0.925 |
| ≤ 0.3 | 38 | 1908 | 19.9 | 1.00 (reference) |  |  | 1.00 (reference) |  |
| > 0.3 | 4 | 255 | 15.7 | 0.77 (0.26 – 2.24) | 0.625 |  | 0.57 (0.20 – 1.64) | 0.297 |

^a^ Adjusted for: admission disease, SOFA Score at enrollment, as fixed coviariates; ECMO, Surgery, number of catheters inserted on the same patient and days of catheterization as time-varying covariate.

^b^ For 1 unit increment.
