# Supplementary material for: Incidence of asymptomatic catheter-related thrombosis in intensive care unit patients: a prospective cohort study
Source: Ann Intensive Care. 2023 Oct 19;13:106. doi: 10.1186/s13613-023-01206-w (PMC10587047; doi:10.1186/s13613-023-01206-w)
Supplement: Supplementary file 4 — Additional file 4: Table S6. Characteristics of CRT patients divided by patients for whom the physician decided to start anticoagulation vs. patients not anticoagulated. [file 13613_2023_1206_MOESM4_ESM.docx]

|  | Overall *  (n = 32) | Anticoagulation  (n = 17) | No Anticoagulation  (n = 15) | P-value |
| --- | --- | --- | --- | --- |
| **ICU Admission data** |  |  |  |  |
| Age (years) | 62 [51 - 72] | 61 [53 - 71] | 66 [43 - 73] | 0.509 |
| Sex (female) | 13 (41%) | 6 (35%) | 8 (53%) | 0.513 |
| BMI (kg/m^2^) | 25.4 [22.3 - 27.6] | 25.5 [22.5 - 27.5] | 25.2 [22 - 28.1] | 0.416 |
| SOFA Score | 3 [3 - 6] | 4 [3 - 6] | 3 [3 - 6] | 0.684 |
| Charlson Comorbidity Index | 2 [1 - 3] | 2 [2 - 3] | 2 [0 - 4] | 0.875 |
| ICU Admission Reason |  |  |  |  |
| Medical | 27 (84%) | 16 (94%) | 11 (73%) | 0.098 |
| ARDS COVID | 6 (19%) | 4 (24%) | 2 (13%) | 0.456 |
| Septic Shock | 8 (25%) | 4 (24%) | 4 (27%) | 0.838 |
| ARDS | 4 (13%) | 4 (24%) | 0 (0%) | 0.018 |
| Cardiac Arrest | 3 (9%) | 1 (6%) | 2 (13%) | 0.468 |
| Acute Liver Failure | 2 (6%) | 1 (6%) | 1 (7%) | 0.927 |
| Others ^a^ | 4 (13%) | 2 (12%) | 2 (13%) | 0.879 |
| Surgical | 5 (16%) | 1 (6%) | 4 (27%) | 0.098 |
| Trauma | 2 (6%) | 0 (0%) | 2 (13%) | 0.074 |
| Others ^b^ | 3 (9%) | 1 (6%) | 2 (13%) | 0.698 |
| **CRT day of diagnosis data** |  |  |  |  |
| Hct (%) | 29 [26 - 35] | 27 [25 - 32] | 31 [27 - 35] | 0.103 |
| Platelets (10^3^/mm^3^) | 174 [114 - 229] | 193 [112 - 241] | 144 [112 - 190] | 0.421 |
| INR | 1.14 [1.06 - 1.21] | 1.06 [1.03 - 1.2] | 1.14 [1.08 - 1.26] | 0.349 |
| aPTT Ratio | 0.89 [0.84 - 0.98] | 0.9 [0.83 - 0.98] | 0.89 [0.84 - 0.99] | 0.771 |
| Fibrinogen (mg/dL) | 476 [357 - 632] | 479 [365 - 702] | 473 [344 - 530] | 0.190 |
| D-dimers (mg/dL) | 6025 [2077 - 14656] | 4676 [1938 - 16391] | 11620 [2110 - 14303] | 0.861 |
| Creatinine (mg/dL) | 1 [0.8 - 1.4] | 1.2 [0.8 - 2.3] | 0.8 [0.6 - 1.3] | 0.173 |
| PaO_2_/FiO_2_ | 230 [168 - 305] | 183 [158 - 264] | 249 [200 - 363] | 0.069 |
| MAP (mmHg) | 80 [75 - 92] | 81 [72 - 88] | 80 [75 - 100] | 0.291 |
| ECMO | 0 (0%) | 0 (0%) | 0 (0%) |  |
| N. Catheters inserted | 2 [1 - 2] | 2 [1 - 2] | 1 [1 - 2] | 0.699 |

**Table 6**

Characteristics of CRT patients divided by patients for whom the physician decided to start anticoagulation vs. patients not anticoagulated

* Excluding patients already fully anticoagulated at CRT diagnosis.

*CRT*, Catheter-Related Thrombosis, *IR,* Incidence Rate; *IRR,* Incidence Rate Ratio; *CI*, Confidence Interval; *pd*, patient-days; *BMI*, Body Mass Index, *SOFA*, Sequential Organ Failure Assessment; *ICU*, Intensive Care Unit; *ARDS*, Acute Respiratory Distress Syndrome; *COVID*, Coronavirus Disease; *ECMO*, Extracorporeal Membrane Oxygenation; *Hct*, Hematocrit; *INR*, International Normalized Ratio; *aPTT*, partial thromboplastin time; *PaO_2_/FiO_2_*, ratio between arterial oxygen partial pressure and oxygen inspired fraction ; *MAP*, mean arterial pressure.
